# Stakeholder perceptions of political and economic factors influencing vaccination in two States with a high burden of zero-dose children in Nigeria

**DOI:** 10.1093/heapol/czag010

**Published:** 2026-01-27

**Authors:** Tanimola Makanjuola Akande, Oladimeji Akeem Bolarinwa, Adekunle Ganiyu Salaudeen, Maysoon Dahab, Olatunde Adesoro, Alhadi Khogali, Samy Ahmar, Tahlil Ahmed, Sostine Makunja, Catherine R McGowan, Nada Abdelmagid

**Affiliations:** Department of Epidemiology and Community Health, University of Ilorin, 1515, P.M.B, Ilorin, Kwara State 240003, Nigeria; Department of Epidemiology and Community Health, University of Ilorin, 1515, P.M.B, Ilorin, Kwara State 240003, Nigeria; Department of Epidemiology and Community Health, University of Ilorin, 1515, P.M.B, Ilorin, Kwara State 240003, Nigeria; Department of Infectious Disease Epidemiology and International Health, Faculty of Epidemiology and Population Health, London School of Hygiene and Tropical Medicine, Keppel Street, London WC1E 7HT, United Kingdom; BOOST Project, Save the Children International Nigeria, Plot 512 Cadastral Zone B09, Behind NAF Conference Centre, Kado, Abuja, Federal Capital Territory 900108, Nigeria; Global Policy Advocacy & Research, Save the Children UK, 1 St John's Lane, London EC1M 4AR, United Kingdom; Global Programmes, Save the Children UK, 1 St John's Lane, London EC1M 4AR, United Kingdom; Global Programmes, Save the Children UK, 1 St John's Lane, London EC1M 4AR, United Kingdom; Global Programmes, Save the Children UK, 1 St John's Lane, London EC1M 4AR, United Kingdom; Department of Infectious Disease Epidemiology and International Health, Faculty of Epidemiology and Population Health, London School of Hygiene and Tropical Medicine, Keppel Street, London WC1E 7HT, United Kingdom; Department of Infectious Disease Epidemiology and International Health, Faculty of Epidemiology and Population Health, London School of Hygiene and Tropical Medicine, Keppel Street, London WC1E 7HT, United Kingdom

**Keywords:** zero-dose, immunization, under-immunized, policy, economy, barriers, coordination, implementation

## Abstract

Globally, an estimated 22.7 million children are unimmunized or ‘zero-dose’ (ZD), with 3.1 million in Nigeria. The political and economic environment plays a critical role in influencing the number of ZD and under-immunized children. We explored stakeholder perceptions of the political and economic context of vaccination services in Kano and Lagos States, two Nigerian States with a high number of ZD children. We conducted stakeholder mapping, followed by key informant interviews with 84 State, local, and community informants responsible for or influential in immunization. Transcripts were analyzed using a reflective thematic framework approach. We describe the multi-level network of domestic and international actors characterizing Nigeria's immunization policymaking and implementation landscape. Respondents perceived a strong and mutual political commitment by all actors involved in routine immunization. The pivotal role of local influencers further reinforced this commitment, from traditional to religious leaders, to improving uptake in challenging settings. Knowledge of national policies, and thus, perception of their adequacy in addressing under-immunization, was weakest among participants working at the local and community levels. Other reported barriers to policy implementation included bureaucratic delays in fund disbursement, outdated policies, slow dissemination of policies to local levels, and inadequate policy provisions for funding and staffing at the local level. To enhance equitable immunization coverage in Kano and Lagos, our findings suggest a need for meaningful engagement of community actors in policy development, timely policy revisions, and the establishment of mechanisms for expediting fund disbursements and addressing funding shortfalls at the local levels.

Key messages

- There is limited evidence on how political and economic factors shape immunization service delivery in Nigeria, especially in States with high numbers of zero-dose children.
- While national and sub-national actors perceive strong political commitment to immunization by all stakeholders, local- and community-level actors are often excluded from policy development and adaptation.
- Inadequate and untimely funding availability at local levels and outdated or poorly disseminated policies hinder routine immunization service delivery.
- Policymakers and influential non-governmental actors should embed community participation in policy processes, accelerate the revision of outdated policies, and establish efficient, reliable mechanisms for channelling timely and adequate funding to the frontlines of immunization service delivery.

## Introduction

Nigeria’s national immunization policy aims to ‘provide immunization services and potent vaccines free to all populations at risk’ (National Primary Health Care Development Agency 2013) through the Expanded Programme on Immunization (EPI) introduced in 1978 (Ophori et al. 2014). Three levels of government—national, State, and local—are expected to share responsibility for the formulation and execution of policies related to the primary healthcare system. Specifically, the federal government is responsible for vaccine procurement, immunization guideline development, and technical direction to sub-national governments, as well as technical support through the National Primary Health Care Development Agency (NPHCDA). State and local governments are primarily responsible for managing infrastructure and logistics to deliver routine immunization (RI) services.

Policy-wise, in 2005, Nigeria adopted the Reach Every Ward strategy to improve immunization coverage (Akwataghibe et al. 2019). In 2018, the government set a target to reduce the number of zero-dose (ZD) children in Nigeria by 80%, and launched a ‘Big Catch-Up Campaign’ in 2023 (World Health Organization 2023b). In 2021, and following a 20% drop in immunization coverage between 2014 and 2016, Nigeria launched the Accelerated Action for Impact initiative aimed at reducing maternal and child deaths (Ibeh 2021).

Achieving EPI immunization targets is not only influenced by technical factors, but also by the political and economic context in which key policies are operationalized (Gavi Zero-Dose Learning Hub 2024). Economic factors often influence political actions related to immunization. For instance, Nigeria’s 2023 annual budget saw a 42.6% increase, raising the health sector’s share to 5.57% of the total budget, up from 4.76% in 2022 (Development Research and Projects Center 2022). Despite this increase, the proportion of the health budget remains significantly below the Abuja Declaration, which set a target of allocating 15% of the total budget to health for African Union member states. Although Gavi, the Vaccine Alliance’s immunization budget for Nigeria saw a 40.9% increase in 2023, challenges related to the adequacy, sustainability, and efficiency of immunization financing persist (Development Research and Projects Center 2022). A 2014 literature review highlighted several economic and political factors potentially influencing RI in Nigeria, including the focus on polio eradication rather than RI, insufficient and geographically inequitable budget allocations, spending inefficiencies, frequent administrative changes, and the politicization of relevant senior appointments (Anyene 2014).

While risk factors for ZD and under-immunized children in Nigeria are well-documented, research on the successes and failures of interventions, including bottlenecks, remains fragmented and primarily focuses on vaccination campaigns and polio eradication efforts (Mahachi et al. 2022). In particular, there is limited understanding of the political and economic factors thought to critically influence the development and implementation of immunization programmes worldwide, and in Nigeria, including in settings with a high proportion of ZD children (Ifedilichukwu 2022). A 2024 political economy analysis in Nigeria focused on factors that influenced data use for immunization programmes. There has not been a political economy analysis focused on the overall immunization programming cycles in Nigeria; this is especially important given policy changes since 2018 (Gavi Zero-Dose Learning Hub 2024). There have been several relevant studies: these included a 2014 study that examined immunization policymaking and governance (Ophori et al. 2014), and a 2019 study that examined immunization policy and implementation structures, as well as policy adoption processes (Akwataghibe et al. 2019). Earlier studies examined overall health policy-making in Nigeria (Uneke et al. 2010), mainly focusing on financing mechanisms for immunization (Wonodi et al. 2012, Chukwuogo and Onwujekwe 2013, Erchick et al. 2017, Alkenbrack et al. 2018).

This study aims to describe the perceptions of key immunization stakeholders on the political and economic factors that influence vaccination services, focusing on two Nigerian States, Kano and Lagos, both of which have a high number of ZD children.

## Methods

### Study design

We conducted a qualitative study using key informant interviews (KIIs) from March to May 2024. Prior to the interviews, we performed stakeholder mapping to identify relevant groups and potential participants. Stakeholders were initially identified through a mapping exercise and continued to evolve through the research team’s network and those identified during the interview process.

### Study setting

We conducted the study in Kano and Lagos States, both with Local Government Areas (LGAs) with a high number of ZD children, defined as infants who have not received the first dose of diphtheria, tetanus, and pertussis-containing vaccine by the end of their first year of life (Gavi Staff 2024).

In Lagos, two mainland LGAs, Alimosho (11 wards) and Ikorodu (19 wards), with populations of 3 082 900 and 1 000 000, respectively, were included in the study. While Alimosho is predominantly urban, with many slums, Ikorodu LGA has some rural and remote areas, including riverine settlements. As of 2021, Alimosho LGA had >35 000 ZD children, the highest absolute number for any LGA in Nigeria, while Ikorodu is one of the 100 LGAs with the highest number of ZD children in Nigeria (Gavi Zero-Dose Learning Hub 2023).

In Kano State, two LGAs—Ungogo (11 wards) and Gezawa (11 wards)—with populations of 369 657 and 282 069, respectively, were studied. Both LGAs are in the central senatorial zone of the State, with Ungongo predominantly metropolitan while Gezawa is mainly rural with some peri-urban communities. Kano State is currently ranked sixth among the most burdened ZD States in Nigeria, with the heaviest burden in rural LGAs, accounting for 58 496 ZD children. Gezawa, a rural LGA, alone accounts for 8592 ZD children, while Ungongo, a semi-urban LGA, is home to 12 250 ZD children (Development Research and Projects Center 2025). The study populations were selected by Save the Children, Nigeria’s Federal Ministry of Health (FMOH), and the NPHCDA, based on the burden of ZD and population profiles, to implement a multi-year programme to improve equitable immunization coverage.

### Study participants

We interviewed immunization stakeholders at the national, State, local land community evels. At the national level, this included representatives from institutions funding, supporting, and managing immunization and developing policies relevant to Kano and Lagos, such as the FMOH, United Nations (UN) agencies, donors, and national and international non-governmental organizations (NGOs). At the State, LGA and community levels, participants included individuals responsible for managing and implementing immunization programmes, social, cultural, and religious leaders, and members of civil society organizations. We also engaged representatives from relevant government ministries, such as Finance, Planning and Budget, and Women’s Affairs.

We engaged community mobilizers who were already part of the existing immunization mobilization structures of the selected LGAs. Because of the trust and familiarity with the communities and political stakeholders, the community mobilizers supported the research in participant identification, selection, and consent. We purposively selected political stakeholders by the geographical areas where their influence and interests are exerted (community/health facility/LGAs/State). Ultimately, the purposively-selected respondents reflected the State, LGA, and community political structures in immunization in Kano and Lagos.

### Data collection

Our research team conducted advocacy visits to the administration and immunization managers of the State Primary Healthcare Boards and LGA officials. The boards gave administrative approval while local immunization officers and managers at LGA level facilitated community entry and mobilization for the study. We recruited two State coordinators (in each of Kano and Lagos) and 22 research assistants. They were trained for 4 days in March 2024 to conduct qualitative data collection in each State. The data collection training included the study’s background, study methods, data collection tools and methods, responsible and ethical conduct of research, data quality assurance, and security information. The study objectives were shared and discussed during the training, which included: the identification of existing policies, protocols, and governance frameworks for increasing immunization coverage; existing bottlenecks in immunization governance; and key partners, collaborators and influencers in the delivery of Nigeria’s immunization policies

We used three versions of translated KII guides for State, LGA, and community stakeholders. Each interview guide was translated into Yoruba and Hausa for Lagos, and Hausa for Kano. These versions reflected the particularities of the political stakeholders at each level. The local investigators and experts in Hausa and Yoruba validated translated versions of the interview guide. We conducted back-translation into English to validate the original translation. The interview guides were pre-tested on the last day of training, with corrections and necessary adjustments made based on feedback received.

Data collection in both States started in March 2024 and was concluded in May 2024. The interviews were conducted in Hausa and Yoruba by paired research assistants, with one acting as a moderator and the other as a note-taker.

During the interview recording and transcription, a participant ID was created in the format ‘stakeholder status/gender/State/LGA/interview number’ to ensure anonymity.

Written informed consent was obtained from each respondent in the local language they spoke fluently, with the help of the community mobilizers. After participant permission was obtained, the interviews were audio-recorded with two Android phones (with or without audio-enhancer microphones, depending on the extent of background noise). The observation notes taken were used to enrich the recorded interviews.

Interviews were transcribed and then translated into English. Transcripts were stored in a secure and password-protected e-cloud (OneDrive). The data will be stored for 5 years.

### Data analysis

We used NVivo software (Lumivero 2018) to organize the data.

We analysed the data in three steps. Firstly, we familiarized ourselves with the data by reading and re-reading the transcripts to keep track of significant emerging ideas. Secondly, we generated initial codes using the study objectives as the initial coding framework. The code was then applied to the entire dataset by labelling data extracts with relevant codes and noting any potential patterns or connections between elements that could feed a later theme. Lastly, we sorted the codes into themes by looking at the list of codes, and collated the codes into broader themes by adapting a reflective thematic framework to analyse the data (Clarke and Braun 2017), and adopted the Problem-Driven Political Economy Analysis Framework (Harris 2013) to organize findings in three thematic areas: (i) structural features, i.e. contextual features of the political and financial landscape; (ii) actors’ agency and interactions that include the relevant individuals and organizations involved, their motivations, and how they interact with each; and, (iii) perceived barriers, and solutions to policymaking and policy implementation in ZD settings.

### Ethical clearance

Ethical clearance was obtained from the authors’ institutes, the National Health Ethical Review Committee, and Kano and Lagos States’ Primary Health Care Boards.

## Results

### Study participants

We conducted KIIs with 84 participants, including 16 at the state level, 32 at the LGA level, and 36 at the community level. Table 1 shows the distribution and categories of the key informants by level and by State.

**Table 1:** Number, distribution, and categories of study participants by level and by State.

Level	Participant type		Number of participants
	Lagos	Kano	
National and State	Government employees (*n = 5*) Non-governmental employees (*n = 2*)	Government employees (*n = 5*) Non-governmental organizations (*n = 4*)	16
Local Government Area	Government employees (*n = 18*)	Government employees (*n = 14*)	32
Community-based	Religious leaders (*n = 4*) Community leaders (*n = 5*) Traditional leader/healer (*n = 4*) Women’s leaders (*n = 2*) Youth leaders (*n = 2*)	Religious leaders (*n = 5*) Civil society and non-governmental organizations (*n* *=* 3) Traditional/district leaders (*n = 9*) Community leaders (*n = 1*) Youth leaders (*n = 1*)	36

**Figure 1:**
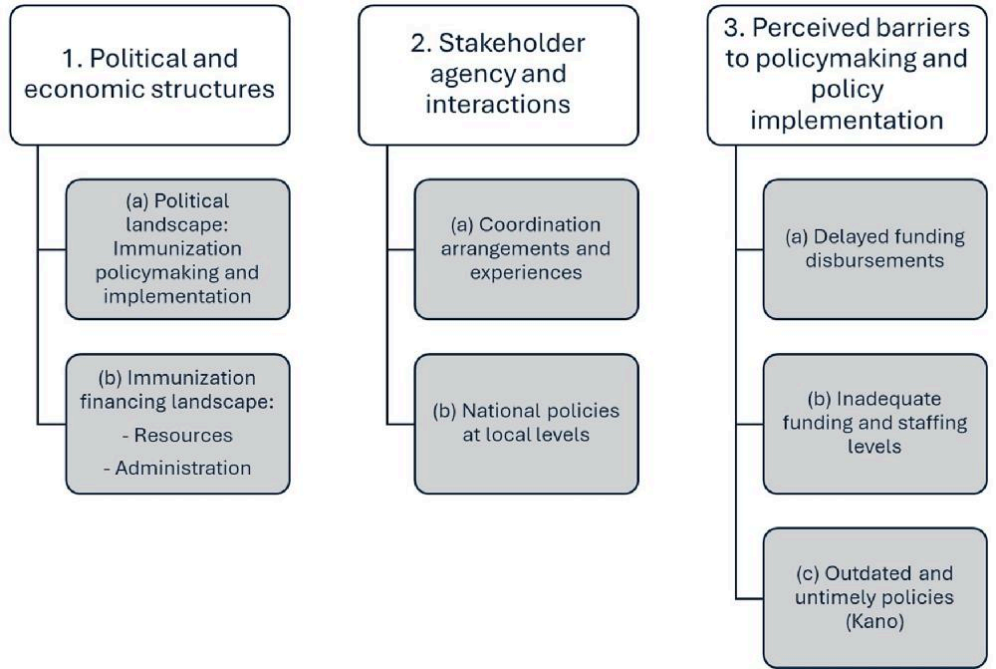
Key sub-themes organized by the components of the Problem-driven Political Economy Analysis Framework.

We organized the main result subthemes across three components (see Fig. 1): (i) political and economic structures; (ii) stakeholder agency and interactions; and (iii) perceived barriers to policymaking and implementation.

#### Political and economic structures: immunization policymaking and implementation

**Figure 2:**
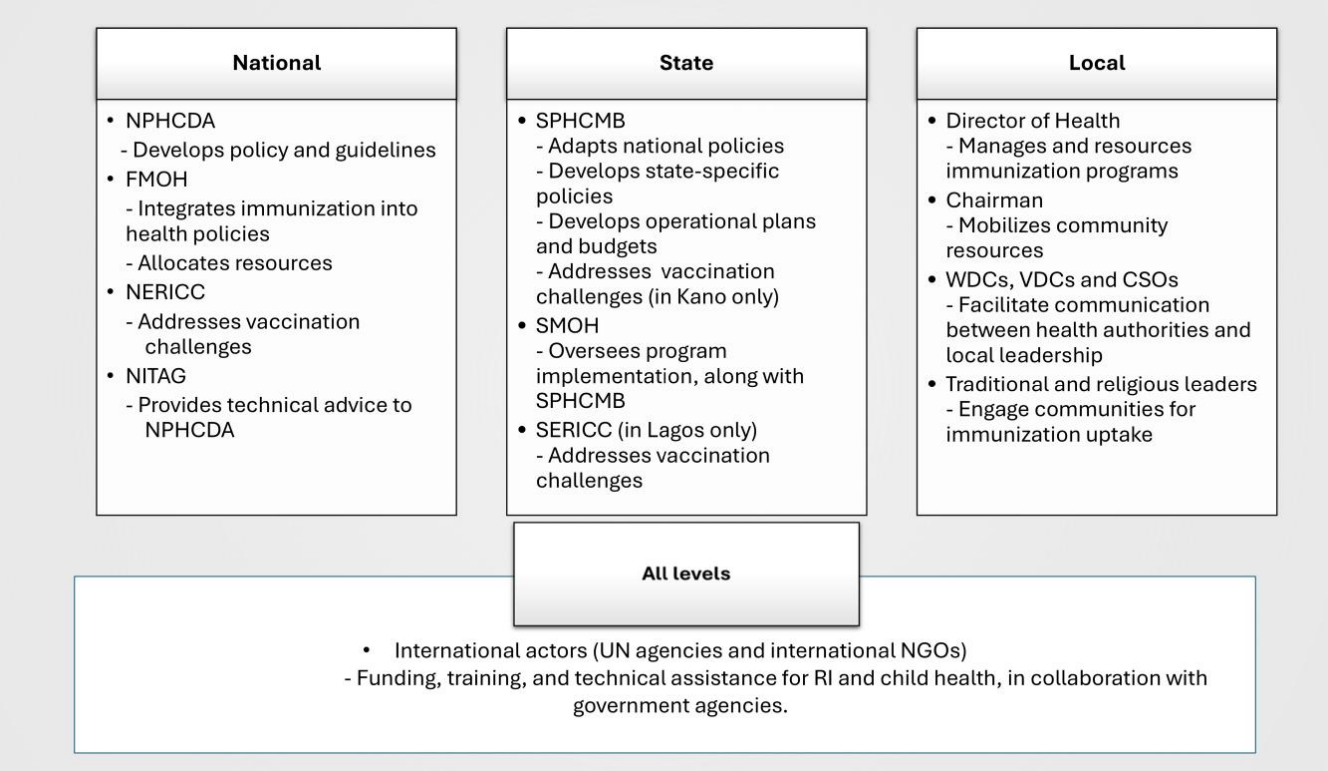
Main reported immunization policymaking and implementation actors in Nigeria.

Participants described the immunization policy landscape as decentralized and shaped by interactions among a network of actors at national, State, and local levels (see Fig. 2). In high ZD-burden settings, the Emergency Routine Immunization Coordination Centre at national (NERICC) and Lagos State (SERICC) levels, and the Kano State Primary Health Care Management Board (SPHCMB), were reported as being responsible for addressing immunization challenges. At local levels, no specific structure or authority was reported.

According to participants, national actors were responsible for setting policies, providing technical assistance, enhancing coordination, addressing immunization challenges, and allocating resources. The EPI programme is part of the NPHCDA and is supported by a National Immunization Technical Advisory Group (NITAG), an independent group of experts who provide technical guidance on vaccines and vaccination. Specifically, the NPHCDA was described as central for developing immunization policies and guidelines. The FMOH, in collaboration with the NPHCDA, incorporates immunization initiatives into broader health strategies and ensures effective resource allocation. To enhance coordination, respondents reported that NERICC and SERICC are tasked with addressing challenges related to low or inequitable vaccination coverage.

Participants reported that State-level governments were primarily responsible for adapting national policies into local contexts, defining State-specific policies, and developing operational plans and budgets. Specifically, the SPHCMB is responsible for immunization and adapts national guidelines to local contexts, including immunization policies such as outreach programmes and vaccine stock management. Participants mentioned that the SPHCMB developed State-specific policies, including a school-based immunization policy in Kano and health staff recruitment policies in Lagos. SPHCMBs also develop operational plans and budgets, overseeing the implementation of immunization programmes in conjunction with the State Ministry of Health.

‘*In Kano, the State Primary Healthcare Board are the key influencers.*’ (KII, Partner, Kano)

At the local level, participants reported that LGAs are vital in implementing immunization policies.

‘*The local government provides an enabling environment, the infrastructure, the structure, the personnel, and unlimited access to the population…the local government is in charge of all of this.*’ (KII, LGA stakeholder, Alimosho, Urban, Lagos)

In both States, the Director of Health was seen as key to ensuring effective management and resourcing of immunization programmes, while the Local Government Chairman played a pivotal role in mobilizing community resources.

‘*… around the governance, we have the commissioner of health who leads all the processes and now we also have the head of the agency especially the head of the State Primary Healthcare Management Board, and then his directors who are also working on immunization and other program officers, so it is in that line.*’ (KII, Partner, Kano)

Also at the local level, participants reported that community leaders and other influential members served as gatekeepers for community engagement, building trust and driving immunization uptake. Local structures, such as the Ward Development Committees (WDCs), Village Development Committees (VDCs), and civil society organisations (CSOs), provide a communication link between health authorities and community members.

‘*The policy [the government] put in place: firstly, they go out, they give people information and also use the local community members because they believe, the people living in an area will know the place better. If [the government] needs something from here, we (living in the area) are the people who know the terrain…*’ (KII, Community stakeholder, Ikodoru, Periurban, Lagos)

Across both States, traditional leaders, including district heads and religious leaders, were described as the main link between immunization implementers and communities. These included State-specific local structures; in Kano, this included the Kano Emirate Council Committee on Health, while in Lagos, these were trusted community figures such as the king and local parents.

‘*….apart from the government is now the traditional leadership: we have the Kano emirate council committee on health. It’s also instrumental when it comes to child health and routine immunization because they have their own structure up to the community level … that are supporting primary health care in the State.*’ (KII, Partner, Kano)

‘*When the new Kabiyesi came, he was very supportive… Kabiyesi is the major stakeholder for now, whenever we go to him, he always rises to support us.*’ (KII, Community stakeholder, Peri-urban, Ikorodu)

Participants strongly agreed that continuous engagement with local actors, especially local leadership, was essential to building trust and driving immunization uptake.

Across all levels, international partners like the World Health Organization (WHO), the United Nations Children’s Fund (UNICEF), the African Field Epidemiology Network (AFENET), Save the Children, and many others were reported to provide support through funding, training, and technical assistance. They collaborate with government agencies to support interventions in RI and child health, and their contributions wield significant influence at all levels.

‘*Well, for Lagos State … we have just the WHO, UNICEF, we have M-RIGHTS. We also have CHAI, and, of course, Save the Children that are coming on board.*’ (KII, State stakeholder, Lagos)

‘*We also have the State team leaders of the various partners, that is, the State team leaders of AFENET, the State team leader of WHO, and the State team leader for UNICEF—they are the main drivers. The key partners [are] AFENET, STOP [Program from] CDC, we have WHO, we have UNICEF, we also have SOLINA, we have CORE Group, we have eHealth Africa, we have Chigari foundation. At LGA level in Gezawa we have Red Cross, they are trying. They are all working towards having zero dose in the country*’. (KII, Partner, Kano)

#### Political and economic structures: immunization financing landscape

The financing and administration of RI in Nigeria are reported to be intertwined through partnerships among government agencies, international actors, and the private sector.

##### Financial resources

Participants described Nigeria’s funding for immunization in Nigeria as being supported by a mix of government and international actors. Participants reported a lack of robust data on the financial contributions of the different stakeholders.

Nonetheless, participants highlighted, largely in favourable terms, the central and highly influential financing role of international actors, such as the UN, international NGOs, and private philanthropic organizations. However, it was also noted that such was the extent of international partner support for immunization that it would be difficult with the current financing ecosystem to implement, let alone sustain, immunization services without external financial and technical assistance. The extent to which this has hindered or facilitated outreach to ZD communities was not described directly, but ZD-specific initiatives were often described as being linked to international donor financing.

‘*The major influencers are the international development partners because their financial contributions are essential. Without them, we might not be able to carry out the program.*’ (KII, Community stakeholder, Ungogo, Urban, Kano)

Respondents also mentioned a ‘basket fund’ where all stakeholders contribute based on a memorandum of understanding (MoU). This fund supports the execution of RI activities, although LGAs contribute little financially, focusing instead on coordination. In Kano State, a specific funding arrangement was described by a participant:

‘*There was an MoU between Kano State government, Bill and Melinda Gates Foundation, and Dangote Foundation for RI in the State. It outlined each party’s financial contributions. Now, with improved [vaccination] coverage, the State government funds all RI activities.*’ (KII, State stakeholder, Kano)

##### Financial administration

As with funding contributions, participants reported that financial administrative responsibilities for components of the immunization programme are also divided. This further enhances the importance of partnerships for the delivery of RI in Nigeria. The federal government, through the NPHCDA, handles most vaccine procurement costs, supported by the WHO and UNICEF, and Gavi, which works through UNICEF’s procurement unit. The vaccines are distributed to States that finance their delivery and work with local authorities to ensure they reach health facilities. NGOs and development partners support logistics, service delivery, and health-worker training, particularly for vaccine administration, managing adverse events following immunization and incorporating new vaccine recommendations.

‘*With immunization costing and financing, some parts are covered by the government, while some parts are covered by donor partners. NGOs and other services support in different areas, like vaccination, donations, logistics, and technical capacity building of health workers. There’s a lot of institutional collaboration, with federal, State, and local governments working together.*’ (*KII, State stakeholder, Lagos)*

Each year, the SPHCMB creates an operational plan and budget for RI, which is integrated into the broader State budget. Once the plan is approved, the ministry of finance disburses funds for immunization activities.

Respondents also highlighted that LGAs contribute to immunization financing, while organizations like the WHO and UNICEF focus on funding specific aspects of RI.

‘*From what I know, WHO is providing resources for logistics, and UNICEF supports social mobilization. These funds are designated for specific activities and cannot be used elsewhere.*’ (KII, Partner, Kano)

#### Stakeholder agency and interactions: coordination arrangements and experiences

Respondents offered contrasting views about the efficiency of coordination and clarity of roles and responsibilities across tiers of government. Some participants described the current system as supporting ‘seamless coordination’ and unifying immunization delivery, while others described centralized immunization coordination as limiting the efficiency and effectiveness of decision-making at local levels.

At the national level, coordination was described as top-down, with NPHCDA collaborating with the FMOH and donor agencies to set the overall direction of RI. State and local levels then implement these activities, with technical support from both local and international partners. As one respondent noted:

‘*At the top, there is the National Primary Health Care Development Agency and the Federal Ministry of Health; these organizations direct and coordinate all immunization activities in the country, in collaboration with partners and donor agencies. At the State level, we have the State Ministry of Health, the State Primary Health Care Development Agency, and at the local government level, we have the Department of Health.*’ (KII, State stakeholder, Kano)

Participants offered contrasting views, even within the same State, on the effectiveness of coordinating national policies through State and local structures. Some participants in Kano described a smooth and efficient coordination mechanism.

‘*We have a smooth process of getting policies from the national level to the State and forward to the LGAs. The coordination of RI is mostly done by the NPHCDA, working hand in hand with the State’s Primary Health Care Boards.*’ (KII, Partner, Kano)

In contrast, several respondents, particularly in Kano, felt that the decision-making for policies and funding at the national level contributed to programmatic challenges and delays. Many of them suggested moving key coordination responsibilities to local levels to improve decision-making.

‘*Primary healthcare should operate from the lower levels—planning, implementation, monitoring, everything. The federal government brings complexities.*’ (KII, Partner, Kano)

In addition, a few respondents perceived that policymaking was overly centralized and lacked the flexibility needed to adapt to the specific needs of different regions or LGAs. The complexity and bureaucratic nature of policymaking were cited as obstacles to quick decision-making and effective local-level implementation.

‘*Nigeria is a complex system, operating federalism, most of the control is at the centre… primary health care is supposed to be a very lower-level kind of… system… If you ask me today, to take on this, I will exclude the federal government from planning, and monitoring, anything to do with immunization, because, before a policy is transmitted down to the lower level, it will take a lot of time. So, we should give routine immunization to the lower level, especially, the local level*’ (KII, Partner, Kano)

Participants described positive instances of effective coordination among local-level actors, including international actors. For example, one participant praised the effective collaboration between health coordinators, partners, and local teams in Gezawa LGA.

‘*At Gezawa LGA, we are working as a team. From the Health Coordinator to the assistants, everyone is trying their best. We work with partners like WHO, UNICEF, New Incentives, and our State team, all of whom are instrumental in helping us carry out our work.*’ (KII, Partner, Kano)

Regarding coordination between governmental and international stakeholders, participants across all tiers reported the existence of focal points at the State and local levels to track each international partner’s contributions and their alignment with the specific needs of the community or State. This coordination is often formalized through a terms of reference or MoU that outlines each partner’s responsibilities.

‘*We have a focal person for partner coordination. Any partner coming into Kano goes through this focal person for discussions on what kind of support they have and in which areas. This prevents repetition and ensures that partner contributions are well-coordinated.*’ (KII, State stakeholder, Kano)

According to participants, successful coordination between governmental and international partners can also be attributed to the assignment of a focused support area, such as logistics, social mobilization or capacity building, for each international actor. Some participants reported that this arrangement reduced day-to-day operational challenges.

‘*When it comes to coordination, we really do not have any challenges. The rules are clearly defined. For example, we know that UNICEF takes care of social mobilization and vaccine logistics, WHO handles logistics during activities, and other partners provide technical and financial support. The coordination has been quite easy because of this clear understanding.*’ (KII, State stakeholder, Lagos)

Once more, the role of local governance structures and traditional and religious leaders was highlighted as integral to enhancing coordination. This was particularly important for engaging community members and addressing challenges arising at the community level.

‘*We have the health workers themselves, and I am also part of the committee. I involve my ward heads and other community groups in decision-making around health or facility issues. We discuss and resolve problems, and sometimes I get suggestions that I never thought of.*’ (KII, Community stakeholder, Gezawa, Peri-urban, Kano)

#### Stakeholder agency and interactions: national policies at local levels

State-level participants believed that there were adequate policies for childhood immunization in place. In contrast, those working at the LGA and community levels found it challenging to articulate specific national immunization policies for settings with high numbers of ZD children. Still, most LGA and community-level respondents expressed positive views about the RI programme, noting increased community acceptance of immunization in recent years.

Local-level participants felt more familiar with local processes than with national policies and strategies focused on ZD children or on immunization more broadly. For example, while most respondents understood immunization services were free, they were unsure if this was a formal policy.

‘*For immunization, I don’t think they collect money. From what I know, I think it’s free. So, I don’t think they collected money from them. However, I don’t know if it’s from the Lagos State government or federal. I didn’t know about that one, but I know that it’s free from health centre.*’ (KII, Community stakeholder, Ikodoru, Periurban, Lagos)

Even without clear knowledge of specific policies for ZD children, respondents acknowledged the government’s and NGOs’ efforts to ensure that all children are immunized.

‘*I don’t know what the policy is but all I know time to time the health care workers do call us and brief us about any development not only on routine immunization but all aspects of health care services… we have seen changes … in the past, we use to have a lot of people who reject vaccination but now we don’t have such cases.*’ (KII, Community stakeholder, Gezawa, Periurban, Kano)

‘*Although this is not my profession, but all I know government and NGOs are doing a lot of things to make sure every child is vaccinated.’* (KII, Community stakeholder, Ungogo, Urban, Kano)

Among the national policies for ZD communities mentioned by participants from Ikorodu LGA (Lagos) and Ungogo LGA (Kano), was an understanding that national policies required RI services to be provided weekly in all health facilities, with the frequency of specific immunization days adjusted to local needs. Other respondents reported a policy that ensures no child is denied vaccination, even if only one child is present at a scheduled vaccination session.

‘*National guidelines clearly state that even if only one child is brought for vaccination, the vial should be opened for that child. The policy’s central goal is to ensure that every child in the country is vaccinated against preventable diseases.*’ (KII, Partner, Kano)

In Kano, several respondents were aware of initiatives by the NPHCDA and partners like UNICEF to reach ZD and under-immunized children through outreach services in hard-to-reach communities.

‘*There is [a] policy which states that outreach services should be provided in hard-to-reach communities; and these are the places where you get zero dose, so I think the NPHCDA knows about this and that is why they come up with the idea of outreach. And there are facilities where RI is being provided daily; and in my opinion all these are policies aimed at eliminating zero dose.*’ (KII, Partner, Kano)

Also in Kano, some participants reported not knowing many details of the national Zero Dose Reduction Plan, launched in 2022, but felt it was a significant step in addressing the large number of ZD children in the State.

‘*…in terms of absolute numbers, Kano leads the country in zero-dose children. In 2022, the National [government] came up with a strategy called [Zero Dose Reduction Plan].*’ (KII, Partner, Kano)

While some participants were aware of a policy to intensify outreach to ZD children, they lacked clarity about who set the policy and how it is implemented.

‘*With the recent mapping of zero-dose children, outreach was intensified to focus on these children. I’m not sure if it’s from the National [government], but they recruited people specifically for this.*’ (KII, Partner, Kano)

Some participants mentioned local coordination processes, e.g. outreach by and coordination with Volunteer Community Mobilizers (VCMs). Specifically, they positively noted the role of regular reconciliation meetings as effective for tracking unvaccinated children.

‘*At the end of each month, the ward head meets with the health facility to reconcile their records of children born and children vaccinated. If any child is missed, the ward head works with the caregivers to ensure they bring the child for immunization.*’ (KII, Partner, Kano)

Community stakeholders from Ungogo, Kano, also highlighted the critical role of VCMs, who worked with community leaders to track and vaccinate under-immunized children.

‘*Yes, there are steps taken to track these children in the community. It involves the use of the junior leaders and the VCMs who work for UNICEF. Also, in the facility there are a few staff that are involved. The community leader has the records of all those that were born, she follows the record to know if a child is immunized or not. Most of the time the VCM works with community leader. VCMs enter the house while the community leaders stay with the men and have a discussion.*’ (KII, Community stakeholder, Ungogou, Urban, Kano)

#### Perceived barriers to policymaking and policy implementation: delayed funding disbursements

Despite an overall positive perception of the coordination mechanisms in place for RI, participants in both Lagos and Kano noted a major concern: delays in releasing funds for immunization activities. Although vaccines are free to children and caregivers at LGA facilities, fund availability for operational activities like vaccine stock collection and transportation was significantly affected by delays in budget releases and complicated bureaucratic processes.

‘*It takes too long for funds to be released due to the bureaucracy involved.*’ (KII, State stakeholder, Kano)

Several partners in Kano noted that the main issue was not insufficient funding but rather the slow bureaucratic processes involved in accessing these funds. One respondent noted that by April, funding for the year had not yet been released, causing delays in maintaining essential services.

‘*Even though there’s a budget, the process to access it is cumbersome, causing delays.*’ (KII, Partner, Kano)

‘*While the budget itself isn’t the issue, the real problem lies in how long it takes for funds to move from the national to State level, which creates significant delays.*’ (KII, Partner, Kano)

‘*Sometimes the budget is not the problem, but before the money is released from the National level down to the States, it takes time; there is always delay [in] the releasing of funds.*’ (KII, Partner, Kano)

Respondents reported that this lag negatively affected the timely execution of RI activities, impacting service delivery across States.

‘*Before funds are released, activities might have already started, meaning we are often forced to begin with insufficient funds. The government is committed, but the delay in disbursement remains a major challenge.*’ (KII, State stakeholder, Kano)

#### Perceived barriers to policymaking and policy implementation: Inadequate funding and staffing levels

At the LGA level, several respondents in Lagos and a few in Kano reported inadequate funding at the local level for implementing RI programmes effectively.

‘*[Funding is] inadequate, highly inadequate, … [it is] one thing is for the fund to be in the budget, another thing is for the fund to be released, another thing is for the fund to get to where it’s really needed and to be used for the purpose for which it’s meant for.*’ (KII, Partner, Lagos)

‘*It is not sufficient, but we are managing to ensure services don’t stop. From the little we receive, we manage to pay temporary staff and support logistics.*’ (KII, Local stakeholder, Gezawa, Kano)

Several respondents called for improved accountability mechanisms to ensure that allocated funds were used efficiently and reached the areas where they were most needed.

‘*Efficient utilization of resources can be achieved through direct payment systems and better accountability mechanisms.*’ (KII, State stakeholders, Kano)

Some, especially in Lagos, attributed the shortfall to competing health services at the LGA level and the lack of dedicated funding and staff for immunization.

‘*There are no specific personnel for immunization. The same staff handling malaria and other [health] services are responsible for RI.*’ (KII, Partner, Lagos)

They felt this contributed to a lack of prioritization of RI services and vice versa.

‘*Well, funding at the State and LGA level, like I said, there’s no budget line for routine immunization. So, what happens is, it’s not prioritized, no. What happens is that there is a general health budget and whatever you get from it, that’s what you work with it*’. (KII, State stakeholder, Lagos)

‘*I think another way is to let the [LGA] chairman know that they should prioritize immunization services at the LGA level. We should let the [LGA] chairman know the importance of immunization to the children and mothers at the LGA level.*’ (KII, Local stakeholder, Alimosho, Urban, Lagos)

In contrast, a few participants in Kano noted that immunization was a priority for State and local governments, with sufficient funding for RI implementation.

‘*Yes, immunization is among the first services that the State and local governments prioritized.*’ (KII, Local stakeholder, Gezawa, Periurban, Kano)

‘*The State government is reviewing it to see how they can increase [the incentive paid to caregivers for vaccinating their children]. 1000 Naira is not adequate for such an activity. For other activities, I can say that the funds are adequate.*’ (KII, Local stakeholder, Ungogo, Urban, Kano)

#### Perceived barriers to policymaking and policy implementation: outdated and untimely policies (Kano)

In Kano, a few respondents raised concerns about outdated policies that hindered effective RI delivery, particularly for reaching ZD or under-immunized populations. They noted that these policies did not reflect current challenges and were slow to be reviewed or updated. Some respondents noted that the rigidity of older policies made it difficult to implement innovative solutions or outreach strategies to address the unique circumstances of hard-to-reach populations.

‘*Some of those documents are kind of obsolete—obsolete in the sense that when you develop a policy document, you need to review it over time to see whether it is actually in tandem with the current realities, or you need to revise or you need to update certain parts of it.*’ (KII, Partner, Kano)

Participants noted that policy review processes were infrequent, and when updates did occur, they were not promptly communicated to the lower levels, where the actual RI activities occur. This lag between policy revisions at the federal or State levels and their transmission to LGAs and communities created a disconnect between policy intent and actual practice.

‘… *in terms of revision and updating of these documents, sometimes it is done at the national level, but then getting it finalized, disseminated to the lower level where the service delivery is actually taking place, sometimes it doesn’t happen. At other times, it tends to linger.*’ (KII, Partner, Kano)

## Discussion

The immunization policymaking and implementation process in Nigeria includes a complex network of multi-level actors. Most participants perceived a strong political commitment to RI facilitated by effective collaboration with international stakeholders at national, State, and local levels. This may reflect recent developments such as the inclusion of ZD priority actions within the National Strategy for Immunization and Primary Healthcare System Strengthening in 2020, informed by a detailed analysis pinpointing 100 LGAs in 18 States that account for 1.5 million of Nigeria’s 2.2 million ZD children, developed through joint workshops and meetings with stakeholders (World Health Organization 2023a). Similar findings were reported by a study in 2019, where State and LGA officials in Ogun State perceived that immunization is a national priority, supported by Gavi and other multilateral organizations, and that clear policies and implementation structures are in place (Akwataghibe et al. 2019). These findings indicate that the multitude of actors involved are mutually committed to RI in Nigeria and are largely well-coordinated. This is manifested in tangible efforts at local levels to address under-immunization, which are visible to local-level authorities and community-based actors and influencers in Kano and Lagos.

Our study highlighted perceptions of positive collaboration among all stakeholders. At the local level, stakeholder interactions were generally perceived as effective and productive, with local authorities and community influencers playing a vital role in building trust and driving immunization uptake. Similar findings were reported in the 2019 study in Ogun State, where respondents noted that successful policy adoption largely depended on cooperation from community leaders (Akwataghibe et al. 2019).

Despite this, actors at the local level were less familiar with national policies than with local RI processes. It was noted that influential local actors, who are key to immunization uptake, do not participate in policy formulation, perhaps explaining the lack of familiarity with national policies at the local level. This suggests a gap in involving local actors in policy development. This is also consistent with our other finding of delays in the timely cascading of new immunization policies to local levels in Kano. A review of the Nigeria immunization programme in 2013 also revealed an over-centralization of policymaking and governance (Ophori et al. 2014). A 2009 study on evidence-based health policymaking in Nigeria identified a similar disconnect between policymakers and implementers, reducing local ownership (Uneke et al. 2010). While our study found a strong commitment to RI locally, a missed opportunity exists to involve local stakeholders in shaping policies that address community-specific challenges. We recommend meaningful engagement of local actors and community members in policy development and revision to ensure suitability, buy-in, and timely communication, all of which will improve service provision.

Respondents reported key barriers to RI implementation overall, which could be especially challenging in settings with high levels of under-immunization. These included bureaucratic delays in fund disbursement, outdated policies, slow policy dissemination to local levels, and insufficient local funding and staffing, particularly in Lagos. These limitations highlight opportunities for enhancing immunization governance in Nigeria. The late release of immunization funds from national to local levels seems to be a longstanding issue in Nigeria, reported in previous studies as early as 2000 (Ophori et al. 2014), through to 2011 (Wonodi et al. 2012, Chukwuogo and Onwujekwe 2013), 2016 (Erchick et al. 2017), and as recently as 2018 (Alkenbrack et al. 2018). While earlier studies reported that the delayed release of funds caused vaccine shortages (Wonodi et al. 2012, Chukwuogo and Onwujekwe 2013, Ophori et al. 2014), funding delays in our study were reported to have mainly had repercussions on the day-to-day operation of services, such as vaccine transportation to facilities. A study using interviews with State and local government officials in Niger State reported similar findings, with funding gaps hampering vaccine collection, supervision, and outreach sessions (Erchick et al. 2017). In the short-term, State policies for emergency funds can be developed to bridge operational funding gaps for vaccine pick-up and logistics.

There were contrasting views of the adequacy of funding levels, with some participants pointing to negative impacts of funding gaps at local levels, particularly in Lagos. While earlier studies in Nigeria reported that an inadequate domestic budget for immunization and vaccines is allocated at the national level (Chukwuogo and Onwujekwe 2013; Ophori et al. 2014), our findings indicate that national and State funding is perceived as sufficient. Still, funding gaps appear at local levels, particularly in Lagos. Participants attributed this shortfall to competing demands on the LGA health budget, where there are no earmarked domestic immunization funds. As of 2018, Nigeria was piloting reforms at federal, State, and local levels to accelerate the implementation of the National Health Act, aimed at increasing and optimizing domestic health resources at the facility level (Alkenbrack et al. 2018)—such initiatives should be ramped up to ensure bottlenecks are addressed. Increasing domestic resource mobilization is particularly important, considering shrinking global funding for childhood immunization (Sunny and Santhosh 2025). We also recommend ring-fencing immunization budgets and dedicated human resources at the LGA level to mitigate the issues of competing demands on the local health budget and workforce.

Finally, we recommend using insights from this study to inform the design of policy and advocacy interventions. We shared the findings and recommendations with key stakeholders in Nigeria during a validation workshop in May 2024. This was used to guide the design of a multi-year intervention to increase equitable vaccination coverage in the study areas.

### Study strengths and limitations

This study allowed us to explore the specific challenges in areas with a high ZD burden. This focus is critical, as Nigeria continues to struggle with equitable coverage. An extensive stakeholder mapping process guided participant selection, and snowball sampling helped identify relevant local actors who might otherwise have been overlooked. However, we did not review policy or financial documents, preventing us from cross-verifying participant reports with other sources - a key limitation given the potential for social desirability bias in the responses.

## Conclusions

Nigeria’s immunization policymaking and implementation process involves a complex network of multi-level actors. Study participants highlighted a strong political commitment to RI. There were contrasting views on the adequacy of existing policies for addressing under-immunization, with most local actors unfamiliar with national policies. While community influencers were seen as vital to building trust and promoting immunization efforts, local actors were underrepresented in policy formulation. Effective collaboration was reported, particularly at the local level, and this was thought to be facilitated by a clear distribution of responsibilities and focused areas to which international partners can contribute. However, key barriers such as bureaucratic delays in fund disbursement, outdated policies and slow policy dissemination to local levels in Kano, and insufficient local funding and staffing—especially in Lagos—were reported.

### Recommendations

We recommend meaningfully engaging local stakeholders in policy development and revisions to ensure suitability, buy-in, and timely communication. We recommend accelerating reforms under the National Health Act to address LGA and facility-level funding bottlenecks, establishing State-level emergency funds to bridge operational funding gaps, and ring-fencing immunization budgets detailing sources for budget contributions at the LGA level, to mitigate competing demands on health resources.

## Data Availability

All relevant data are within the manuscript.

## Reflexivity statement

Our authorship team reflects a commitment to inclusivity across gender, seniority, and geographical representation. The first three authors are senior professors based in Nigeria, while the group is equally balanced along gender lines. The team is led by the last author, who is a female Sudanese national based in London.
